# Neuropsychological and Psychosocial Functioning of Children with Perinatal HIV-Infection in The Netherlands

**DOI:** 10.3390/v13101947

**Published:** 2021-09-28

**Authors:** Stefanie E. M. van Opstal, Emma J. Dogterom, Marlies N. Wagener, Femke K. Aarsen, Harald S. Miedema, Pepijn D. D. M. Roelofs, Linda C. van der Knaap, Pieter L. A. Fraaij, Kim Stol, André B. Rietman, Eric C. M. van Gorp, Annemarie M. C. van Rossum, Elisabeth M. W. J. Utens

**Affiliations:** 1 Center of Expertise Innovations in Care, Rotterdam University of Applied Sciences, 3015 EK Rotterdam, The Netherlands; m.n.wagener@hr.nl (M.N.W.); h.s.miedema@hr.nl (H.S.M.); p.d.d.m.roelofs@hr.nl (P.D.D.M.R.); 2 Department of Viroscience, Erasmus MC University Medical Center, 3015 GD Rotterdam, The Netherlands; e.vangorp@erasmusmc.nl; 3 Department of Pediatrics, Division of Infectious Diseases and Immunology, Erasmus MC University Medical Center-Sophia Children’s Hospital, 3015 GD Rotterdam, The Netherlands; e.dogterom@erasmusmc.nl (E.J.D.); l.vanderknaap@erasmusmc.nl (L.C.v.d.K.); p.fraaij@erasmusmc.nl (P.L.A.F.); k.stol@erasmusmc.nl (K.S.); a.vanrossum@erasmusmc.nl (A.M.C.v.R.); 4 Department of Psychosocial Care and Psychology, Princess Maxima Center for Pediatric Oncology, 3584 CS Utrecht, The Netherlands; F.K.Aarsen@prinsesmaximacentrum.nl; 5 Department of Child and Adolescent Psychiatry, Erasmus MC University Medical Center, 3015 CN Rotterdam, The Netherlands; a.rietman@erasmusmc.nl (A.B.R.); l.utens@levvel.nl (E.M.W.J.U.); 6 Research Institute of Child Development and Education, University of Amsterdam, Levvel, 1018 WS Amsterdam, The Netherlands

**Keywords:** perinatally HIV-infected children, long-term outcomes, neuropsychological and psychosocial development

## Abstract

Advances in antiretroviral treatment improved the life expectancy of perinatally HIV-infected children. However, growing up with HIV provides challenges in daily functioning. This cross-sectional cohort study investigated the neuropsychological and psychosocial functioning of a group of perinatally HIV-infected children in the Netherlands and compared their outcomes with Dutch normative data and outcomes of a control group of uninfected siblings. The children’s functioning was assessed with internationally well-known and standardized questionnaires, using a multi-informant approach, including the perspectives of caregivers, teachers, and school-aged children. In addition, we explored the associations of socio-demographic and medical characteristics of the HIV-infected children with their neuropsychological and psychosocial functioning. Caregivers reported compromised functioning when compared to Dutch normative data for HIV-infected children in the areas of attention, sensory processing, social-emotional functioning, and health-related quality of life. Teachers reported in addition compromised executive functioning for HIV-infected children. A comparison with siblings revealed differences in executive functioning, problems with peers, and general health. The concurrent resemblance between HIV-infected children and siblings regarding problems in other domains implies that social and contextual factors may be of influence. A family-focused approach with special attention to the child’s socio-environmental context and additional attention for siblings is recommended.

## 1. Introduction

Due to the introduction of combination Antiretroviral Therapy (cART), HIV infection is nowadays considered a chronic disease rather than a lethal disease [1]. As a result, the first generation of children living with a perinatally-acquired HIV infection is able to reach school age and adolescence. As HIV affects perinatally infected children in a crucial period of development, the long-term effects of growing up with HIV warrant investigation and careful monitoring. It is increasingly recognized that children with HIV are at risk for neuropsychological and psychosocial problems [2,3].

Neuropsychological functioning is a broad concept, including both basic cognitive processes (i.e., perception and sensory processing) and higher-order cognitive processes (i.e., executive functioning). The use of cART has been shown to decrease the incidence of HIV encephalopathy in perinatally infected children from more than 20% to under 2% [4]. Nevertheless, neuropsychological deficits remain present in perinatally infected children, even with an adequate viral suppression. A systematic review showed a prevalence of neuropsychological deficits in HIV-infected children (0–18 years), varying from less than 20% to up to 90% in some subdomains. This suggests that HIV affects neuropsychological functioning particularly in certain areas, such as accuracy of information processing and working memory [2,5]. The exact pathway from an HIV-infection to neuropsychological deficits is a complex one, as direct effects of the virus on the brain may be complemented by long-term effects of medication, periods of hospitalization, and environmental challenges such as low socio-economic status, which can play a crucial role in child development [6,7].

Psychosocial functioning encompasses as main domains behavioral functioning, emotional well-being, and social competence, and is of utmost importance for a child’s daily functioning and quality of life [8]. Regarding psychosocial functioning, the available studies showed that HIV-infected children experience more problems when compared with the general population, with higher levels of anxiety, depression, and conduct problems [9,10]. Multiple family-related factors and contextual factors may be involved, as the lives of many HIV-infected children are affected by parental illness and death, poverty, social isolation, and stigmatization.

In addition, considering the potential neuropsychological and psychosocial challenges, it is not surprising that HIV-infected children may have difficulties in school functioning. Studies report on learning problems, drop-outs, the need for extra support at school, and enrollment in fulltime special education [11,12,13]. Education is universally recognized to be essential for children’s development; for HIV-infected children, education is specifically important to provide a sense of normalcy and to reduce risk behavior [14].

In summary, it is increasingly recognized that HIV-infected children face challenges in daily functioning. Few studies so far have specifically addressed the neuropsychological and psychosocial functioning of perinatally HIV-infected children of a broad age range, covering the total school period. Until now, no studies have investigated the neuropsychological and psychosocial functioning of school-aged children with HIV using a multi-informant approach; that is: including the perspectives of caregivers, teachers, and school-aged children themselves. Most studies focused on a selected group of children of a small age range, did not include an appropriate control group, or were conducted in a context where access to healthcare services and cART was limited [5,15,16,17,18,19].

We aimed to bridge a gap in knowledge through a systematic study of the neuropsychological and psychosocial functioning of a large Dutch pediatric HIV-population, with a group of siblings as control group. This is clinically important because identifying neuropsychological and psychosocial problems in an early phase will enable the implementation of attuned interventions. Consequently, if during our study a child’s individual results indicated the need for further extensive clinical diagnostic neuropsychological assessment, he or she was referred to a pediatric neuropsychologist for assessment of neuropsychological or psychosocial deficits.

Primary aim: To compare the neuropsychological and psychosocial functioning of perinatally HIV-infected children living in the Netherlands with Dutch normative data and with data of their uninfected siblings from the perspectives of caregivers, teachers, and children.

Secondary aim: To explore associations of socio-demographic and medical characteristics of HIV-infected children with their neuropsychological and psychosocial functioning.

## 2. Materials and Methods

### 2.1. Study Design

A cross-sectional cohort study was conducted at the Erasmus University Medical Center-Sophia Children’s Hospital in Rotterdam, The Netherlands, between April 2014 and July 2016. Ethical consent for this study was given by the Medical Ethics Committee (MEC-2013-424), of Erasmus MC Rotterdam, the Netherlands.

### 2.2. Study Population

A total of 62 HIV-infected children received medical treatment at Erasmus MC-Sophia Children’s Hospital at the time of the study initiation. Eligible for inclusion were perinatally HIV-infected children (5–18 years) who had been living in the Netherlands for more than two years. Exclusion criteria were: an actual opportunistic infection of the central nervous system or diagnosed intellectual disability. Eligible children and their caregivers were invited to participate in our study, together with their HIV-negative siblings and teachers.

Caregivers were excluded if they had no command of the English or Dutch language, or if they had been taking care of the HIV-infected child for less than two years. HIV-negative siblings (5–18 years) were eligible for inclusion if they had lived in the same household with the HIV-infected child for more than two years. If multiple siblings were eligible for inclusion in one family, the sibling with the smallest age gap to the HIV infected child was chosen. Within each family, a maximum of one sibling was included to prevent burden for caregivers.

### 2.3. Study Procedure

Caregivers and/or HIV-infected children were invited to participate in the study by their HIV nurse or pediatrician after a consultation at the hospital. Written informed consent was obtained from all caregivers, teachers and children aged 12 years and older.

### 2.4. Neuropsychological and Psychosocial Assessment

To assess neuropsychological and psychosocial functioning, we administered internationally well-known questionnaires with adequate psychometric properties, age-attuned normative data and a multi-informant approach (parent/caregivers’ reports, teachers’ reports and self-reports). For all total and subscale scores on these questionnaires, we determined if the results were in the clinical range, indicating a problem in the area or areas in question. The clinical range was defined according to the available standards for each questionnaire [20,21,22,23,24,25,26] (see Appendix A).

#### 2.4.1. Neuropsychological Assessment

##### Executive Functioning

Executive functioning was tested with three parallel versions of the Behavior Rating Inventory of Executive Function (BRIEF): caregivers’ report, a teachers’ report, and a self-report (for 11 years and older). The BRIEF evaluates day-to-day problems related to executive functioning, resulting in the Behavior Regulation Index (consisting of three scales), the Metacognition Index (five scales), and the combined Global Executive Composite score (all scales). The Dutch version of the BRIEF covers the age-range of 5–18 years, and has shown adequate psychometric properties [22].

##### Sensory Processing

Sensory processing was measured with two versions of the Sensory Profile (SP-NL): the Short Sensory Profile (SSP) for caregivers (of children 5–12 years old), and the Adolescent/Adult Sensory Profile (AASP) for self-completion (11–18 years) [23,24]. Dutch normative data were not yet available for the AASP. Therefore, we derived normative data from a study conducted in Belgium, which made use of the Dutch version of the AASP [27]. Both versions have shown adequate psychometric qualities [23,27,28,29,30].

#### 2.4.2. Psychosocial Assessment

##### Behavioral and Emotional Functioning

Behavioral and emotional functioning were assessed with the Dutch version of the Strengths and Difficulties Questionnaire (SDQ) [31,32]. We used three parallel versions for completion by caregivers, teachers (of children 5–18 years old), and self-report (for >10 years). The Dutch versions of the SDQ have adequate psychometric qualities [33].

##### Health-Related Quality of Life

To evaluate the subjective health and illness of the HIV-infected children as well as any limitations in physical, social, and school functioning, the HRQOL subdomains general health, physical-, and role/social functioning were assessed with the Child Health Questionnaire (CHQ) for caregivers (of children 5–18 years old), and for self-report (11–18 years). The Dutch version of the CHQ has shown adequate psychometric qualities [25,26,34,35].

### 2.5. Indication for Further Extensive Neuropsychological Assessment (NPA)

Since deficits in neuropsychological functioning can result in problems in psychosocial functioning and vice versa, results of both the neuropsychological and psychosocial questionnaires were considered for the indication for further neuropsychological assessment (NPA). Results of the questionnaires were summarized into a standardized report for each child. The report gave an overview of all (sub)scales and indicated scores in the clinical range (see Appendix A).

Each report was structurally discussed among four team members: two senior pediatric clinical (neuro)psychologists, the child’s pediatrician, and the child’s HIV nurse. A referral for further NPA was based on consensus among those four team members. NPA was indicated if there were one or more clinical scores on the neuropsychological questionnaires. In addition, one or more clinical scores on the psychosocial questionnaires, borderline scores on the neuropsychological questionnaires, or information from clinical consultations regarding problems in daily functioning were also discussed to determine the need for referral for NPA.

### 2.6. Socio-Demographic Characteristics

Socio-demographic characteristics (including age, sex, ethnicity, socio-economic status (SES), educational level, adoption status, and home environment) were collected using a standardized questionnaire for caregivers, specifically designed for this study. SES was determined by the caregivers’ occupational level, based on combined information about both highest occupation and highest education. Using the standard coding system of Statistics Netherlands, occupational levels were categorized in low, middle, and high [36]. Ethnicity was divided into three categories: African (both biological parents born in an African country), mixed (one biological parent born in the Netherlands, the other parent abroad), and other. Home environment was divided into three categories: living with at least one biological parent, adopted, and other.

### 2.7. Medical Characteristics

Medical data (including viral load, CD4-count and CDC-nadir) were retrieved from medical records, and blood samples were taken during inclusion by the HIV nurse. Blood samples were already routinely taken for the regular medical care. The Centers for Disease Control and Prevention (CDC) uses a system to classify HIV disease and infection into mutually exclusive categories, ranging from non-symptomatic (N) to clinical conditions defining AIDS (C) [37]. We obtained information regarding CDC-nadir: the lowest level of CDC-classification ever measured.

### 2.8. Statistical Analyses

The sociodemographic characteristics of the total group of included HIV-infected children and the group of siblings were compared in group-level analyses using independent samples’ *t*-tests or Fisher’s exact tests where appropriate. Results on the neuropsychological and psychosocial questionnaires of the group of HIV-infected children were compared with normative data and with the results of the uninfected siblings in group-level analyses. Scores on all questionnaires were summarized into subscale scores and a total score for the SDQ (Total difficulties score) and BRIEF (Global Executive Composite score). Raw scores of the BRIEF were transformed into Z-scores, adjusted for age and sex. Normal distribution of all data was tested with the Shapiro–Wilk test, and linearity was tested with scatterplots. Homogeneity of variance was tested with Levene’s test. Patients’ results were compared with those of the siblings using independent sample *t*-tests or Mann–Whitney U tests. Results of both HIV-infected children and their siblings were compared with normative data using (summary) independent samples’ *t*-tests or Wilcoxon signed rank tests. Two-sided testing was used, and a *p*-value of <0.05 was considered significant.

To explore associations between socio-demographic and medical characteristics of the HIV-infected children and their neuropsychological and psychosocial functioning, univariate analyses were performed between the variables age, sex, ethnicity, SES, adoption status, home environment, CDC-nadir, CD4 count, and viral load at inclusion and:

1. Presence (or absence) of an indication for further NPA;
2. Executive functioning according to the BRIEF Global Executive Composite score of caregivers’, teachers’, and self-report;
3. Behavioral and emotional functioning according to the SDQ total difficulties score of the caregivers’, teachers’, and self-report.

We performed univariate logistic (ad 1) regression or linear regression analyses (ad 2 and 3). Multivariable analyses were not applicable due to the small sample sizes. All statistical analyses were conducted with IBM SPSS Statistics (Version 26) for Windows.

## 3. Results

### 3.1. Participant Characteristics

**Figure 1:**
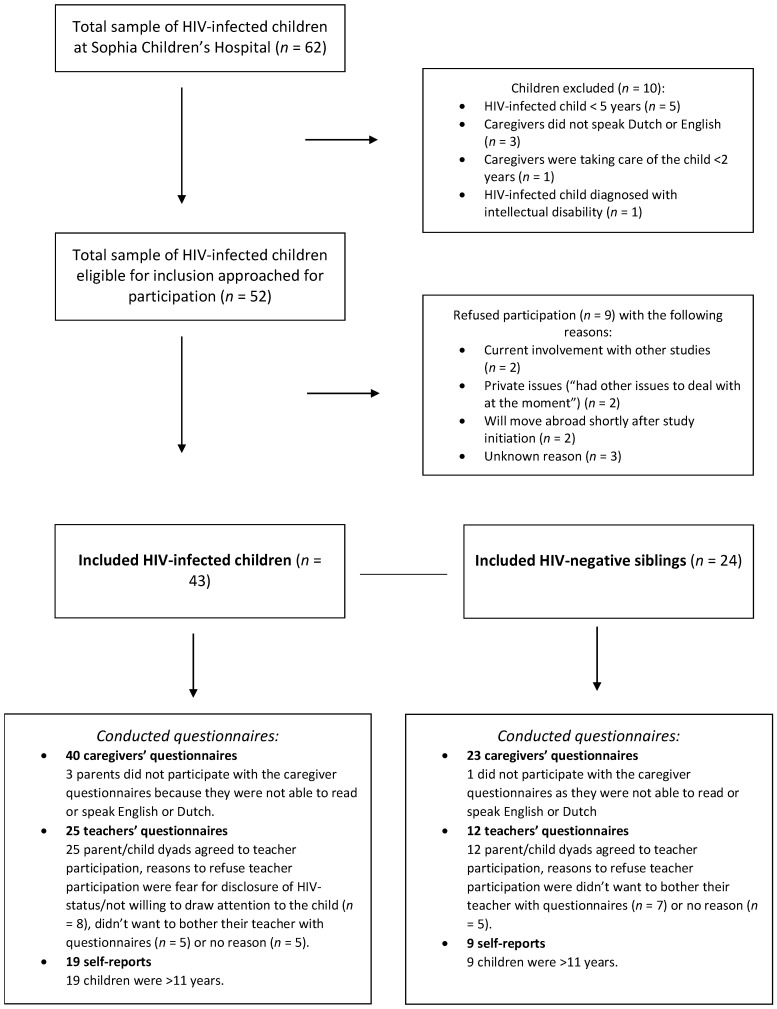
Inclusion flowchart.

Fifty-two HIV-infected children were eligible for inclusion, of whom 43 participated (83%). Non-participants refused participation due to involvement with other studies (*n* = 2), private issues (*n* = 2), intended migration abroad shortly after study initiation (*n* = 2), or an unknown reason (*n* = 3). Regarding the remaining 43 HIV-infected children, 24 had siblings eligible for inclusion. All 24 eligible siblings participated (See Figure 1). A total of 19 HIV-infected children did not have a sibling eligible for inclusion; they were only children or had siblings not meeting the inclusion criteria. When comparing the total group of HIV-infected children (*n* = 43) with the total group of siblings (*n* = 24) in group-level analyses, no significant differences were found on sociodemographic characteristics (Table 1).

For logistical reasons, we included one 19-year-old HIV-infected girl who had not yet made the transition to adult care. Children not born in the Netherlands had moved to the Netherlands and had been living here on average 4.6 years prior to study inclusion (HIV-infected children) or 4.9 years (siblings). All children were enrolled in school—most of them in regular primary or high school. Special education was needed in 23% of the HIV-infected children and 17% of the siblings. All HIV-infected children were treated with cART, and 38 (88%) had an undetectable viral load (<20 copies/ML) at study inclusion.

Regarding the 43 HIV-infected children, we received 40 caregivers’ questionnaires, 25 teachers’ questionnaires, and 19 self-reports. Regarding the 24 siblings, we received 23 caregivers’ questionnaires, 12 teachers’ questionnaires, and 9 self-reports. All invited teachers and all eligible children (>11 years) had responded (Figure 1).

### 3.2. Executive Functioning

#### 3.2.1. Caregiver Reports

Caregivers reported significantly better functioning for the HIV-infected children compared to Dutch normative data on the BRIEF Global Executive Composite score, the Behavior Regulation Index, and four subscales: Inhibit, Emotional Control, Organization of Materials, and Monitor (Table 2). The caregivers also reported statistically better functioning for HIV-infected children compared with the group of siblings on the Behavior Regulation Index and three subscales: Shift, Emotional Control, and Monitor.

#### 3.2.2. Teacher Reports

In contrast to the caregivers, the teachers reported significantly more problems for the HIV-infected children compared to Dutch normative data on the BRIEF Global Executive Composite score, the Metacognition Index, and four subscales: Shift, Initiate, Working Memory, and Plan/Organize (Table 3). In addition, teachers reported significantly more problems for HIV-infected children than for their siblings on the Metacognition Index and two subscales: Initiate and Plan/Organize.

#### 3.2.3. Self-Reports

Scores of HIV-infected children did not show any significant differences with Dutch normative data or with scores of siblings (Appendix B).

### 3.3. Sensory Processing

#### 3.3.1. Caregiver Reports

Caregivers reported significantly more problems for HIV-infected children compared to Dutch normative data on the tactile sensitivity, auditory filtering, and low energy/weak scales. In addition, caregivers reported significantly more problems on the scale movement sensitivity for HIV-infected children when compared to siblings (Appendix C).

#### 3.3.2. Self-Reports

The self-reported scores of HIV-infected children did not indicate problems in sensory processing when compared to normative data. There were no significant differences on the self-reports between HIV-infected children and siblings (Appendix C).

### 3.4. Behavioral and Emotional Functioning

#### 3.4.1. Caregiver Reports

Caregivers reported significantly more problems for the HIV-infected children compared to Dutch normative data on the SDQ total difficulties score and three subscales: emotional problems, hyperactivity/inattention, and problems with peers (Table 4). Compared with their siblings, caregivers reported significantly more problems with peers for HIV-infected children. For siblings, caregivers reported significantly more conduct problems compared to HIV-infected children.

#### 3.4.2. Teacher Reports

Teachers reported no significant differences on the SDQ scores of HIV-infected children when compared to Dutch normative data and siblings (Appendix D).

#### 3.4.3. Self-Reports

Self-reported scores of the HIV-infected children were not significantly different from Dutch normative data and scores of siblings (Appendix D).

### 3.5. Health-Related Quality of Life

#### 3.5.1. Caregiver Reports

Caregivers reported significantly lower scores, indicating more problems, for HIV-infected children on the scales of general health, physical functioning, and role/social limitations due to emotional and behavioral problems, compared to Dutch normative data (Appendix E). Compared to the results of the siblings, caregivers reported significantly more problems for HIV-infected children on the scale of general health.

#### 3.5.2. Self-Reports

Self-reported data were not significantly different from Dutch normative data. When compared with siblings, HIV-infected children reported significantly more problems on the scale of general health.

### 3.6. Reasons for Further Neuropsychological Assessment (NPA)

For 63% (*n* = 27) of the HIV-infected children and 58% (*n* = 14) of the siblings, there was an indication for further neuropsychological assessment. These children had one or more scores in the clinical range according to the standards for each questionnaire (See Appendix A). For HIV-infected children, scores in the clinical range were most frequently found in:

- Caregiver-reported SSP auditory filtering subscale (31%);
- Caregiver-reported SDQ total difficulties score (30%);
- Caregiver-reported SDQ hyperactivity/inattention subscale (25%);
- Teacher-reported SDQ emotional problems subscale (25%);
- Caregiver-reported CHQ role/social limitations due to emotional and behavioral problems subscale (26%).

### 3.7. Associations of Socio-Demographic/Medical Characteristics with Neuropsychological and Psychosocial Functioning

Univariate analyses of the relations between neuropsychological or psychosocial outcomes or indication for NPA, and socio-demographic and medical characteristics of HIV-infected children showed: (1) an association of the caregiver-reported Global Executive Composite BRIEF score with age, indicating more problems on overall executive functioning with increasing age (*p* = 0.043), and (2) an association of the self-reported Total difficulties SDQ score- and SES, indicating more psychosocial problems with lower SES (*p* = 0.034).

### 3.8. Results of Siblings

In addition, caregiver-, teacher-, and self-reported scores regarding the siblings were compared to normative data. Caregivers reported significantly more problems for siblings in several areas: the BRIEF Shift subscale, the SSP subscales Tactile sensitivity, Underresponsive/seeks sensation, and Auditory filtering, the SDQ subscales Emotional problems, Hyperactivity/inattention and Conduct problems, and the Total difficulties SDQ score and the CHQ subscale role/social limitations due to emotional and behavioral problems. The results of both teacher’s reports and self-reports of siblings were not significantly different from normative data.

## 4. Discussion

This cross-sectional cohort study investigated the neuropsychological and psychosocial functioning of HIV-infected children, using a multi-informant approach, including the perspectives of caregivers, teachers, and school-aged children. The results indicate that, according to caregivers’ reports, the included HIV-infected children experienced more problems in their neuropsychological and psychosocial functioning when compared with Dutch normative data, especially in the areas of attention, sensory processing, emotional functioning, contact with peers, and aspects of health-related quality of life. In addition, teachers reported problems for the HIV-infected children in their executive functioning. On the self-reports, the HIV-infected children did not report problems in their neuropsychological or psychosocial functioning when compared to normative data, and only one difference with siblings on a scale of health-related quality of life: general health. When caregiver-reported scores of the HIV-infected children were compared with those of the control group of siblings, differences were less pronounced as when compared with Dutch normative data. This relates to the evident problems we found among siblings as well, particularly in emotional functioning, behavioral functioning, and attention. Furthermore, we found an association of increased caregiver-reported problems for HIV-infected children on executive functioning with age, and an association with increased self-reported psychosocial problems with lower SES.

As the socio-demographic characteristics of HIV-infected children and their siblings were similar, differences in functioning might reflect the (direct) influence of the HIV-infection. The most pronounced differences between HIV-infected and siblings were found in teacher-reported executive functioning, caregiver-reported problems with peers and caregiver-, and self-reported general health. The teacher-reported difference in executive functioning is in line with previous studies that show that this domain of cognitive functioning is particularly compromised in HIV-infected children [5,11,38,39].

Currently, the exact pathophysiological mechanism of how HIV affects the developing brain, compounded by long-term effects of cART, is still unclear. Nevertheless, increasing evidence implies that ongoing neuroinflammation, vascular dysfunction, and hypercoagulability, induced by HIV, may impact the development of the pediatric brain [6]. Previous studies discovered significant damage to the neuronal microstructure, lower gray and white matter volumes, and poorer white matter integrity in HIV-infected children [6,40,41]. These macro- and microstructural changes have been associated with poorer cognitive performance [6,40,41]. In addition, a review revealed that HIV might impact particularly the fronto-striatal system, which is involved in executive functioning [42]. In contrast with previous literature, our study did not reveal associations of executive functioning with medical characteristics, which could be explained by the limited sample size or the little variation in current medical characteristics due to successful treatment with cART. Previous studies found associations between lower CD4 cell count, a history of AIDS-defining diagnoses, and higher peak viral load with compromised executive functioning [43,44]. These studies and our finding that HIV-infected children have more teacher-reported problems in their executive functioning than their siblings, support the hypothesis that the HIV-infection is a contributor to cognitive problems, especially executive functioning. The development of problems in executive functioning is of significant concern for its negative impact on academic learning, successful transition to adulthood, and medication adherence [43,45]. Hence, care providers should be aware of the risk for compromised executive functioning in HIV-infected children, and refer them to a neuropsychologist if problems in this domain arise. Furthermore, the relation with immunological status, which has been revealed in previous studies, underscores the importance of early cART initiation and monitoring [43,44].

Caregivers reported more problems for the HIV-infected children in the area of peer relationships, when compared to siblings and Dutch normative data. These peer problems may be associated with (fear for) HIV-related stigma and disclosure, although the HIV-status of most of the children in our study had not been disclosed at school or to peers. However, this lack of disclosure implies living with a secret, which may contribute to a perception of being different [18]. A further explanation for peer problems among HIV-infected children could be found in associations with neurocognitive problems. Previous studies have found that HIV-infected children were more likely to have peer problems if they presented with neuroimaging abnormalities [46] or encephalopathy [18]. In addition, an association between compromised executive functioning and problems in contact with peers has been described before [47]. It is worth noting that this association could be mediated by problems with inattention or hyperactivity [47,48], which are areas for which caregivers in our study frequently reported problems for HIV-infected children. Another explanation for peer problems may be found in deficits in social cognition, which have been described for HIV-infected adults [49]. Social cognition encompasses the cognitive capacities to facilitate social interaction, and problems in this area could have disabling influences on people’s social communication and relationships [49]. Future research exploring social cognition in HIV-infected children is recommended.

In both caregivers’ reports and self-reports, scores on the general health scale were significantly worse for HIV-infected children when compared to their siblings. The HIV-infected children in our study received adequate treatment and multidisciplinary care. However, given the fact they do have a chronic disease, it can be expected that they have more concerns about their health or that they do not feel as healthy as their siblings. The above finding highlights the importance for HIV-practitioners to be aware of the health-related concerns of both HIV-infected children and their caregivers.

One of the most consistent outcomes of our study is that none of the self-reported scores of the HIV-infected children themselves were significantly different from the normative data, and we found only one difference with siblings (on general health). This lack of statistically significant differences might be explained by the small number of self-reports, although the scores on the self-reports were generally close to normative data. This suggests that HIV-infected children truly did not experience difficulties in their neuropsychological and psychosocial functioning. This outcome might reflect limited awareness of own neuropsychological and psychosocial functioning. As executive functions are necessary to accurately judge one’s performance, deficits in this area may have contributed to decreased self-awareness and may have led to an overestimation of one’s competence [50,51]. Furthermore, the phenomenon of positive illusory bias, where one’s self-reported competence is substantially higher than one’s actual competence, has been associated with problems in executive functioning and attention or hyperactivity deficits [51,52]. Since HIV-infected adolescents are expected to become partners in their own health care and have to deal with challenging medical regimens, it is recommended for HIV-practitioners to be aware that HIV-infected children may have limited awareness of difficulties in neuropsychological and psychosocial functioning.

Besides the clear differences that we found between HIV-infected children and their siblings, another important finding of our study is also that, for both groups, similar psychosocial and neuropsychological aspects were compromised, compared to normative data. For both HIV-infected children and their siblings, caregivers reported emotional problems, problems with hyperactivity or inattention, and problems in sensory processing. This resemblance in problems between HIV-infected children and their siblings implies a major role of social and contextual factors herein.

Many of the included children—both HIV-infected children and siblings—were living with one or both HIV-infected parents. In this respect, they were vulnerable for parental illness and death. A review shows that, in comparison with children from HIV-free families, HIV-affected children are at risk for emotional problems [53]. Potential stressors associated with parental HIV, including stigma and uncertainty about clinical course of parental HIV, could aggravate the negative impact on children. Moreover, other stressful life events were common among our study population, including migration and changes of caregivers, which may further compromise psychosocial and neuropsychological functioning. About 40% of the HIV-infected children and their siblings in this study were adopted children. A previous study in a group of international adoptees showed that specifically attention, sensory processing, and executive functioning were compromised [54]. Furthermore, in our study, approximately 40% of the HIV-infected children and their siblings were living in a family with a low SES. SES affects a wide array of health-related, psychosocial, and neuropsychological outcomes [55]. In our study, we found an association of increased self-reported psychosocial problems with lower SES. This is in line with previous literature, in which low SES has been associated with increased emotional problems, and externalized problems, such as hyperactivity [56]. In addition, a review concluded that children in families of low SES are on average two times more likely to have attention deficit/hyperactivity disorder [57]. Except for SES and age, we did not find associations of socio-demographic and medical characteristics with neuropsychological and psychosocial functioning in our study. Possibly, associations are more subtle or found in specific domains, instead of the composite scores used in our study. These associations should be further explored with larger sample sizes. Nevertheless, healthcare professionals should be aware of the potential risk of all these social and contextual factors for the neuropsychological and psychosocial functioning of both HIV-infected children and their siblings.

Of special concern are the problems in attention that the caregivers often reported for both HIV-infected children and siblings. Caregivers specifically reported problems in the subscales tactile sensitivity and auditory filtering of the SP, which suggest possibly lower thresholds for sensory information, resulting in distractibility and attentional problems. As attention is an important predictor of academic performance, problems in this area could lead to compromised school functioning [58]. It is therefore important for healthcare professionals to monitor the school functioning of HIV-infected children, and to help HIV-infected children, their siblings, and parents navigate through the educational and healthcare system to receive the services they need. Furthermore, future research is recommended to explore the relationship between attention problems and social and contextual factors in HIV-infected children and their siblings.

Caregivers and teachers of the HIV-infected children in our study indicated that part of the problems were found in different areas. Teachers reported more problems in executive functioning for HIV-infected children compared to Dutch normative data, whereas caregivers—remarkably—reported better outcomes compared to normative data regarding executive functioning and relatively more problems regarding emotional and social functioning. To check if this contradiction was not a result of inequalities between children with or without teacher participation, we performed an additional sensitivity analysis. We found no significant differences between both groups in socio-demographic and medical characteristics and between results on the caregivers-reported total difficulties score of the SDQ and Global Executive Composite score of the BRIEF. This suggests that the results truly reflect a difference in functioning as observed by teachers and caregivers. Possibly, the favorable scores of caregivers were influenced by ’response shift’, for which the phenomenon involves a change in internal standards and values after traumatic events [59]. Due to events such as adoption, migration, changes of caregivers, and the often emotional experiences related to the diagnosis and consequences of the HIV-infection of their child, caregivers may have expected worse and be happy with their child’s present functioning. This may have led to an overestimation of their child’s functioning. To note, response shift for executive functioning has been described before in a study using caregivers’ reports of the BRIEF in children after cardiac arrest [60]. A further explanation for the contrast between caregivers’ reports and teachers’ reports is that these findings are related to differences between the home and school environment. School settings might demand greater independence from children than the home environment, where family support might help children to accomplish everyday tasks. Particularly, caregivers of children with a chronic disease could be more caring and providing, and become overprotective [61]. Thereby, they might have accommodated to the child’s condition and may have become accustomed to compensate for the child’s problems in executive functioning, thereby possibly hindering the development of independence at home [62]. Therefore, the school environment might be more challenging regarding executive functioning. Furthermore, teachers could probably compare the executive functioning of the HIV-infected child with that of more peers. Moreover, caregivers might not be aware of a child’s problems in executive functioning because they tend to focus on other areas, such as the child’s emotional and social well-being. While the caregivers’ concerns might have been prompted by the fear for HIV-related stigma, the teachers were mostly unaware of the HIV-infection. In addition, increasing deficits in executive functioning may emerge later in childhood when these functions are expected to mature, which is called ‘growing into deficits’ [63]. This is in line with our finding that caregiver-reported problems increased with the children’s age.

The results of this study must be interpreted with caution. First, given the limited sample size, we may have failed to detect problems in neuropsychological and psychosocial functioning and associations with socio-demographic and medical characteristics. Especially for the teachers’ reports and the self-reports, the numbers of respondents were limited. Although a strength of our study is the inclusion of a control group of siblings with comparable socio-demographic characteristics, the sample size of this control group was small. The extent to which this affected the results of the control group is unknown because the risk of chance findings is higher with a smaller group. This may have resulted in a failure to detect differences. Future research with a larger sample size of the siblings is therefore recommended. Second, several statistical tests were performed on a large number of variables, which could have resulted in a type 1 error. Because of the explorative character of the study, we are less concerned, however, about this possible bias. Third, the cross-sectional design limits the possibility of drawing causal conclusions. Future research would therefore benefit from a longitudinal design containing a larger sample size. Lastly, there are limitations associated with the measurement methods, including reliance on reports from caregivers, teachers, and the HIV-infected children and their siblings themselves. Comprehensive further neuropsychological assessment of both HIV-infected children and their siblings would be helpful in strengthening our findings. Notwithstanding these limitations, this cross-sectional study provides important information regarding neuropsychological and psychosocial functioning of HIV-infected children. The inclusion of a control group of siblings enabled us to explore the possible impact of the HIV-infection with a HIV-negative control group with similar socio-demographic characteristics such as SES, parental HIV, and adoption. Understanding the prevalence and etiology of compromised neuropsychological and psychosocial functioning is essential, as it may impact HIV-infected children’s daily functioning, quality of life, and academic performance. Early detection and intervention might help lower the impact on daily living and prevent deterioration of HIV-infected children and their siblings. A family-focused approach with special attention to a child’s socio-environmental context and additional attention for siblings is recommended.

## Figures and Tables

**Table 1:** Socio-demographic and medical characteristics of patients and siblings.

Variable	HIV-Infected Children (*n* = 43)	Siblings (*n* = 24)	*p*-Value
**Sex child**			0.45 ^1^
Male	46.5%	50.0%	
**Mean age child in years at inclusion**	9.9	9.8	0.98 ^2^
**Ethnicity**			0.18 ^1^
African	46.5%	66.7%	
Mixed	37.2%	16.7%	
Other	16.3%	16.7%	
**Home environment**			0.89 ^1^
Living with at least one biological parent	51.2%	50.0%	
Adopted	37.2%	41.7%	
Other	11.6%	8.3%	
**Born in the Netherlands**	46.5%	45.8%	0.96 ^1^
**SES**			0.65 ^1^
Low	44.2%	37.5%	
Middle	25.6%	37.5%	
High	30.2%	25.0%	
**CDC-nadir**			
N	18.6%		
A	25.6%		
B	18.6%		
C	37.2%		

^1^ Fisher exact test; ^2^ Independent Samples *t* test.

**Table 2:** Results BRIEF caregivers’ report.

	HIV-Infected Children Mean (SD)	Siblings Mean (SD)	Dutch Normative Mean (SD) [22]	*p*-Value ^1^
BRIEF caregivers’ report	*n* = 40	*n* = 23	*n* = 3333	
Global Executive Composite Score	46.0 (10.4)	51.3 (9.3)	50.0 (10.0)	0.012 * ^a^ 0.534 ^b^ 0.054 ^c^
Behavioral Regulation Index	45.1 (11.3)	53.4 (9.2)	50.0 (10.0)	0.002 * ^a^ 0.105 ^b^ 0.005 * ^c^
Metacognition index	46.9 (9.8)	49.8 (9.3)	50.0 (10.0)	0.054 ^a^ 0.935 ^b^ 0.255 ^c^
Inhibit	46.4 (10.9)	51.5 (10.3)	50.0 (10.0)	0.024 * ^a^ 0.480 ^b^ 0.077 ^c^
Shift	46.9 (11.5)	54.1 (10.3)	50.0 (10.0)	0.054 ^a^ 0.049 * ^b^ 0.017 * ^c^
Emotional control	45.1 (9.9)	52.8 (8.1)	50.0 (10.0)	0.002 * ^a^ 0.184 ^b^ 0.002 * ^c^
Initiate	47.2 (9.0)	48.4 (9.0)	50.0 (10.0)	0.076 ^a^ 0.441 ^b^ 0.579 ^c^
Working Memory	51.0 (9.6)	51.6 (8.3)	50.0 (10.0)	0.521 ^a^ 0.441 ^b^ 0.794 ^c^
Plan/Organize	47.4 (9.1)	49.8 (10.1)	50.0 (10.0)	0.099 ^a^ 0.935 ^b^ 0.373 ^c^
Organization of materials	46.3 (9.4)	48.4 (9.7)	50.0 (10.0)	0.021 * ^a^ 0.453 ^b^ 0.388 ^c^
Monitor	44.4 (10.1)	49.8 (9.2)	50.0 (10.0)	0.000 * ^a^ 0.935 ^b^ 0.047 * ^c^

* = *p* < 0.05. ^a^ = patient vs. Dutch normative data. ^b^ = sibling vs. Dutch normative data. ^c^ = patient vs. sibling. ^1^ = Independent-Samples *t* Test. Dutch normative data [22]. A higher score indicates more problems in executive functioning.

**Table 3:** Results BRIEF teachers’ report.

	HIV-Infected Children Mean (SD)	Siblings Mean (SD)	Dutch Normative Mean (SD) [22]	*p*-Value
BRIEF teachers’ report	*n* = 25	*n* = 12	*n* = 941	
Global Executive Composite Score	54.0 (6.5)	49.1 (8.0)	50.0 (10.0)	0.049 * ^a,1^ 0.751 ^b,1^ 0.054 ^c,1^
Behavioral Regulation Index	52.9 (7.1)	50.6 (9.3)	50.0 (10.0)	0.097 ^a,2^ 0.842 ^b,1^ 0.435 ^c,3^
Metacognition Index	54.8 (8.2)	47.8 (7.4)	50.0 (10.0)	0.018 * ^a,1^ 0.454 ^b,1^ 0.017 * ^c,1^
Inhibit	50.9 (7.5)	52.7 (8.9)	50.0 (10.0)	0.662 ^a,1^ 0.358 ^b,1^ 0.526 ^c,1^
Shift	54.8 (8.6)	49.7 (7.9)	50.0 (10.0)	0.018 * ^a,1^ 0.909 ^b,1^ 0.074 ^c,3^
Emotional control	52.0 (8.8)	50.6 (8.7)	50.0 (10.0)	0.210 ^a,2^ 0.842 ^b,1^ 0.672 ^c,3^
Initiate	54.9 (10.4)	47.8 (8.1)	50.0 (10.0)	0.016 * ^a,1^ 0.454 ^b,1^ 0.046* ^c,1^
Working Memory	54.7 (8.2)	49.0 (8.0)	50.0 (10.0)	0.021 * ^a,1^ 0.730 ^b,1^ 0.055 ^c,1^
Plan/Organize	55.5 (8.7)	48.4 (7.9)	50.0 (10.0)	0.007 * ^a,1^ 0.586 ^b,1^ 0.024 * ^c,1^
Organization of materials	51.5 (7.3)	49.5 (7.1)	50.0 (10.0)	0.418 ^a,2^ 0.863 ^b,1^ 0.397 ^c,3^
Monitor	50.8 (9.7)	47.4 (6.5)	50.0 (10.0)	0.678 ^a,1^ 0.373 ^b,1^ 0.275 ^c,1^

* = *p* < 0.05. ^a^ = patient vs. Dutch normative data. ^b^ = sibling vs. Dutch normative data. ^c^ = patient vs. sibling. ^1^ = Independent-Samples *t* Test. ^2^ = Wilcoxon Signed-Ranks Test. ^3^ = Mann–Whitney Test. Dutch normative data [22]. A higher score indicates more problems in executive functioning.

**Table 4:** Results SDQ caregivers’ report.

	HIV-Infected Children Mean (SD)	Siblings Mean (SD)	Dutch Normative Data [21] Mean (SD)	*p*-Value
SDQ caregivers’ report	*n* = 40	*n* = 23	*n* = 300	
Total difficulties score	10.3 (5.3)	11.3 (6.3)	6.7 (5.3)	0.000 * ^a,1^ 0.000 * ^b,1^ 0.485 ^c,1^
Emotional problems	2.5 (2.0)	3.1 (2.4)	1.8 (1.9)	0.025 * ^a,1^ 0.002 * ^b,1^ 0.287 ^c,1^
Conduct problems	1.4 (1.7)	2.0 (1.6)	1.0 (1.4)	0.121 ^a,1^ 0.005 * ^b,2^ 0.044 * ^c,3^
Hyperactivity/inattention	4.5 (2.6)	5.0 (3.0)	2.7 (2.7)	0.000 * ^a,1^ 0.000 * ^b,1^ 0.451 ^c,1^
Peer problems	2.0 (1.6)	1.3 (1.7)	1.1 (1.6)	0.001 * ^a,1^ 0.243 ^b,2^ 0.047 * ^c,3^
Prosocial	8.3 (2.5)	8.4 (1.8)	8.5 (1.5)	0.469 ^a,1^ 0.913 ^b,2^ 0.788 ^c,3^

* = *p* < 0.05. ^a^ = patient vs. Dutch normative data. ^b^ = sibling vs. Dutch normative data. ^c^ = patient vs. sibling. ^1^ = Independent-Samples *t* Test. ^2^ = Wilcoxon Signed-Ranks Test. ^3^ = Mann–Whitney Test. Dutch normative data [21]. A higher score indicates more problems in in behavioral and emotional functioning, except for the prosocial scale where a lower score indicates more problems.

## Data Availability

The data presented in this study are available on request from the corresponding author. The data are not publicly available due to privacy issues.

## Appendix A. Definition of Scores in the Clinical Range

For scores on all (sub)scales of the neuropsychological and psychosocial questionnaires, we indicated if the results were in the clinical range, indicating a problem in this area. Scores in the clinical range were defined according to the available standards for each questionnaire, based on Dutch normative data [20,21,22,23,24,25,26]:

**Questionnaire**	**Clinical Range**	**References**
*Strength and Difficulties Questionnaire (SDQ)*		
- SDQ caregiver report and teacher report for children 5–7 years	- Scores above the 90th percentile (p90)	Mieloo et al., 2012
- SDQ caregiver report, teacher report and self-report for children 8–19 years	- Scores above the 90th percentile (p90)	Goedhart, Treffers, & Van Widenfelt, 2003
*Behavior Rating Inventory of Executive Function (BRIEF)*		
- BRIEF caregiver report, teacher report and self-report for children 5–18 years	>1.5 Standard Deviation (SD) above the mean score, adjusted for age and sex	Huizinga & Smidts, 2012
*Sensory Profile (SP)*		
- SP caregiver report (Short Sensory Profile) for children 5–12 years	>2SD difference from the mean score (with three different mean scores for children 4–5 years, 6–10 years, and 11–12 years)	Dunn & Rietman, 2013
- Adolescent/Adult Sensory Profile (AASP) for self-completion for children 11–18 years	>2SD difference from the mean score	Brown et al., 2007
*Child Health Questionnaire*		
- Caregiver report (CHQ-PF50) for children 5–19 years	>1SD below the mean score	Raat, Bonsel et al., 2002
- Self-report (CHQ-CF87) for children 11–19 years	>1SD below the mean score	Raat, Landgraf et al., 2002

## Appendix B. Results BRIEF Self-Reports

BRIEF Self-Reports

	**HIV-Infected** **Children****Mean (SD)**	**Siblings Mean (SD)**	**Dutch Normative Mean (SD)** [22]	***p*-Value ^1^**
BRIEF self-report	*n* = 19	*n* = 9	*n* = 1687	
Total (Global Executive Composite) Score	50.1 (10.7)	48.3 (13.1)	50.0 (10.0)	0.983 ^a^ 0.618 ^b^ 0.714 ^c^
Behavioral Regulation Index	48.4 (12.0)	49.0 (14.0)	50.0 (10.0)	0.495 ^a^ 0.835 ^b^ 0.911 ^c^
Metacognition index	51.6 (9.3)	48.8 (12.3)	50.0 (10.0)	0.480 ^a^ 0.715 ^b^ 0.500 ^c^
Inhibit	46.7 (10.8)	51.6 (12.0)	50.0 (10.0)	0.158 ^a^ 0.641 ^b^ 0.298 ^c^
Shift	50.7 (10.0)	49.9 (13.1)	50.0 (10.0)	0.748 ^a^ 0.974 ^b^ 0.851 ^c^
Emotional Control	48.7 (10.4)	47.6 (9.4)	50.0 (10.0)	0.585 ^a^ 0.465 ^b^ 0.775 ^c^
Working Memory	52.1 (9.6)	49.6 (9.6)	50.0 (10.0)	0.347 ^a^ 0.895 ^b^ 0.525 ^c^
Plan/Organize	51.2 (9.9)	49.3 (8.6)	50.0 (10.0)	0.615 ^a^ 0.841 ^b^ 0.639 ^c^
Organization of materials	49.1 (7.3)	46.2 (10.0)	50.0 (10.0)	0.680 ^a^ 0.258 ^b^ 0.402 ^c^
Monitor	51.0 (10.3)	52.0 (10.4)	50.0 (10.0)	0.665 ^a^ 0.550 ^b^ 0.813 ^c^
Task Completion	52.4 (8.6)	48.7 (11.6)	50.0 (10.0)	0.304 ^a^ 0.691 ^b^ 0.351 ^c^
^a^ = patient vs. Dutch normative data. ^b^ = sibling vs. Dutch normative data. ^c^ = patient vs. sibling. ^1^ = Independent-Samples *t* Test. Dutch normative data [22]. A higher score indicates more problems in executive functioning.				

## Appendix C. Results Sensory Profile Caregivers’ Report and Self-Report

SSP Caregivers’ Reports

	**HIV-Infected Children Mean (SD)**	**Siblings Mean (SD)**	**Dutch Normative Data Mean (SD) [23]**	***p*-Value**
SSP caregivers’ report	*n* = 32	*n* = 19	*n* = 1257	
Tactile sensitivity	31.0 (3.9)	30.6 (4.6)	33.0 (2.7)	−0.000 *^,a,1^
				0.009 *^,b,2^
				0.769 ^c,3^
Taste/Smell Sensitivity	17.5 (3.5)	18.5 (2.2)	17.8 (2.9)	0.526 ^a,1^
				0.185 ^b,2^
				0.206 ^c,3^
Movement Sensitivity	13.4 (2.2)	14.5 (1.2)	13.7 (1.8)	0.418 ^a,1^
				0.017 *^,b,2^
				0.045 *^,c,3^
Underresponsive/Seeks Sensation	28.3 (4,0)	26.4 (7.3)	29.0 (4.2)	0.360 ^a,1^
				0.009 *^,b,1^
				0.308 ^c,1^
Auditory filtering	20.9 (5.6)	20.7 (5.6)	23.9 (3.9)	0.000 *^,a,1^
				0.000 *^,b,1^
				0.902 ^c,1^
Low energy/weak	27.7 (4.3)	29.3 (1.3)	28.6 (2.4)	0.046 ^a,1^
				0.025 ^b,2^
				0.166 ^c,3^
Visual/Auditory Sensitivity	21.3 (3,6)	19.9 (5.3)	21.4 (2.7)	0.759 ^a,1^
				0.628 ^b,2^
				−0.492 ^c,3^
* = *p* < 0.05. ^a^ = patient vs. Dutch normative data. ^b^ = sibling vs. Dutch normative data. ^c^ = Patient vs. Sibling. ^1^ = Independent-Samples *t* Test. ^2^ = Wilcoxon Signed-Ranks Test. ^3^ = Mann–Whitney Test. Dutch normative data [23]. A lower score indicates that more problems were reported by caregivers/parents.				

AASP Self-Reports

	**HIV-Infected Children Mean****(SD)**	**Siblings Mean (SD)**	**Normative Data Mean (SD)** [27]	***p*-Value ^1^**
AASP self-report	*n* = 19	*n* = 9	*n* = 33	
Low Registration	33.1 (9.0)	31.1 (9.7)	30.5 (6.4)	0.233 ^a^
				0.811 ^b^
				0.607 ^c^
Sensation seeking	42.1 (8.7)	41.8 (9.0)	51.2 (6.8)	0.000 *^,a^
				0.001 *^,b^
				0.927 ^c^
Sensory Sensitivity	32.9 (8.4)	35.4 (12.6)	34.3 (7.0)	0.529 ^a^
				0.715 ^b^
				0.592 ^c^
Sensation Avoidance	31.4 (9.0)	33.7 (10.9)	30.2 (8.4)	0.641 ^a^
				0.309 ^b^
				0.559 ^c^
* = *p* < 0.05. ^a^ = patient vs. normative data. ^b^ = sibling vs. normative data. ^c^ = Patient vs. Sibling. ^1^ = Independent-Samples *t* Test. Belgian normative data [27]. A higher score indicates more problems were reported (self-report).				

## Appendix D. Results SDQ Teachers’ Report and Self-Report

SDQ Teachers’ Reports

	**HIV-Infected Children** **Mean (SD)**	**Siblings** **Mean (SD)**	**Dutch Normative Data** **Mean (SD)** [21]	***p*-Value**
SDQ teachers’ report	*n* = 25	*n* = 12	*n* = 208	
Total score	8.8 (6.0)	6.3 (7.7)	7.6 (6.6)	0.387 ^a,1^ 0.116 ^b,2^ 0.104 ^c,3^
Emotional problems	2.6 (2.4)	1.3 (2.1)	1.7 (2.0)	0.209 ^a,2^ 0.109 ^b,2^ 0.073 ^c,3^
Conduct problems	0.8 (1.1)	0.5 (1.0)	1.0 (1.8)	0.268 ^a,2^ 0.109 ^b,2^ 0.412 ^c,3^
Hyperactivity/inattention	4.0 (3.1)	3.3 (3.4)	2.8 (3.0)	0.061 ^a,1^ 0.937 ^b,2^ 0.386 ^c,3^
Peer problems	1.5 (1.5)	1.3 (2.3)	2.0 (2.2)	0.111 ^a,2^ 0.067 ^b,2^ 0.325 ^c,3^
Prosocial	7.3 (2.2)	7.2 (2.6)	8.1 (2.2)	0.068 ^a,2^ 0.158 ^b,1^ 0.948 ^c,3^
^a^ = patient vs. Dutch normative data. ^b^ = sibling vs. Dutch normative data. ^c^ = patient vs. sibling. ^1^ = Independent-Samples *t* Test. ^2^ = Wilcoxon Signed-Ranks Test. ^3^ = Mann–Whitney Test. Dutch normative data [21]. A higher score indicates more problems in in behavioral and emotional functioning, except for the prosocial scale where a lower score indicates more problems.				

SDQ Self-Reports

	**HIV-Infected Children Mean (SD)**	**Siblings Mean (SD)**	**Dutch Normative Data Mean (SD)** [21]	***p*-Value**
SDQ self-report	*n* = 19	*n* = 9	*n* = 1353	
Total score	9.3 (5.9)	10.6 (6.5)	9.9 (4.9)	0.607 ^a,1^
				0.690 ^b,1^
				0.621 ^c,1^
Emotional problems	2.6 (2.3)	2.3 (1.7)	2.2 (2.0)	0.351 ^a,1^
				0.842 ^b,1^
				0.733 ^c,1^
Conduct problems	1.7 (1.7)	2.1 (2.0)	2.0 (1.5)	0.421 ^a,2^
				0.863 ^b,2^
				0.464 ^c,3^
Hyperactivity/inattention	3.3 (2.3)	4.3 (2.9)	3.8 (2.3)	0.362 ^a,1^
				0.489 ^b,1^
				0.317 ^c,1^
Peer problems	1.7 (1.5)	1.8 (2.3)	1.9 (1.7)	0.686 ^a,2^
				0.511 ^b,2^
				0.724 ^c,3^
Prosocial	8.0 (1.7)	7.9 (1.5)	7.2 (2.3)	0.082 ^a,2^
				0.370 ^b,1^
				0.802 ^c,3^
^a^ = patient vs. Dutch normative data. ^b^ = sibling vs. Dutch normative data. ^c^ = Patient vs. sibling. ^1^ = Independent-Samples *t* Test. ^2^ = Wilcoxon Signed-Ranks Test. ^3^ = Mann–Whitney Test. Dutch normative data [21].				

A higher score indicates more problems in behavioral and emotional functioning, except for the prosocial scale where a lower score indicates more problems.

## Appendix E. Results CHQ Caregiver-Report and Self-Report

CHQ Caregivers’ Reports

	**HIV-Infected Children** **Mean (SD)**	**Siblings** **Mean (SD)**	**Dutch Normative Data** **Mean (SD)** [25]	***p*-Value**
CHQ caregivers’ report	*n* = 40	*n* = 23	*n* = 353	
General health	66.8 (17.4)	76.7 (19.8)	82.9 (13.4)	0.000 * ^a,1^ 0.426 ^b,2^ 0.002 * ^c,3^
Physical functioning	96.3 (13.3)	97.0 (8.0)	99.1 (4.3)	0.004 * ^a,1^ 0.228 ^b,1^ 0.693 ^c,1^
Role/social limitations- emotional behavior	88.6 (22.1)	87.9 (18.8)	97.9 (7.2)	0.000 * ^a,1^ 0.021 * ^b,1^ 0.896 ^c,1^
Role/social limitations- physical	97.1 (9.2)	97.7 (7.8)	95.8 (15.6)	0.610 ^a,1^ 0.307 ^b,1^ 0.781 ^c,1^
* = *p* < 0.05. ^a^ = patient vs. Dutch normative data. ^b^ = sibling vs. Dutch normative data. ^c^ = patient vs. sibling. ^1^ = Independent-Samples *t* Test. ^2^ = Wilcoxon Signed-Ranks Test. ^3^ = Mann–Whitney Test. Dutch normative data [25]. A lower score indicates more problems.				

CHQ Self-Reports

	**HIV-Infected Children Mean (SD)**	**Siblings Mean (SD)**	**Dutch Normative Data Mean (SD)** [26]	***p*-Value**
CHQ self-report	*n* = 19	*n* = 9	*n* = 444	
General health	68.9 (17.0)	83.5 (14.6)	74.6 (15.9)	0.127 ^a,1^
				0.096 ^b,1^
				0.035 *^,c,1^
Physical functioning	93.8 (8.5)	90.5 (23.0)	96.8 (5.4)	0.219 ^a,2^
				0.763 ^b,2^
				0.649 ^c,3^
Role/social limitations-emotional	84.2 (25.2)	87.7 (23.2)	92.3 (16.8)	0.836 ^a,2^
				0.582 *^,b,2^
				0. 774 ^c,3^
Role/social limitations-behavior	94.2 (15.0)	84.0 (27.3)	91.4 (13.7)	0.076 ^a,2^
				0.854 ^b,2^
				0.258 ^c,3^
Role/social limitations-physical	96.5 (10.5)	92.6 (12.4)	96.5 (11.6)	0.076 ^a,2^
				0.854 ^b,2^
				0.288 ^c,3^
* = *p* < 0.05. ^a^ = patient vs. Dutch normative data. ^b^ = sibling vs. Dutch normative data. ^c^ = patient vs. sibling. ^1^ = Independent-Samples *t* Test. ^2^ = Wilcoxon Signed-Ranks Test. ^3^ = Mann–Whitney Test. Dutch normative data [26]. A lower score indicates more problems.				
